# Low Operational Stability of Enzymes in Dry Organic Solvents: Changes in the Active Site Might Affect Catalysis

**DOI:** 10.3390/molecules17021870

**Published:** 2012-02-14

**Authors:** Vibha Bansal, Yamixa Delgado, Marc D. Legault, Gabriel Barletta

**Affiliations:** 1 Department of Chemistry, University of Puerto Rico at Cayey, Cayey, 00736, Puerto Rico; Email: vibha.bansal@upr.edu; 2 Department of Chemistry, University of Puerto Rico at Humacao, Humacao, 00791, Puerto Rico; Email: yami1084@hotmail.com (Y.D.); 3 Department of Physics, University of Puerto Rico at Bayamón, Bayamón, 00959, Puerto Rico; Email: marc.legault@upr.edu

**Keywords:** subtilisin Carlsberg, organic solvents, EPR spectroscopy, enzyme kinetics in organic solvents

## Abstract

The potential of enzyme catalysis in organic solvents for synthetic applications has been overshadowed by the fact that their catalytic properties are affected by organic solvents. In addition, it has recently been shown that an enzyme’s initial activity diminishes considerably after prolonged exposure to organic media. Studies geared towards understanding this last drawback have yielded unclear results. In the present work we decided to use electron paramagnetic resonance spectroscopy (EPR) to study the motion of an active site spin label (a nitroxide free radical) during 96 h of exposure of the serine protease subtilisin Carlsberg to four different organic solvents. Our EPR data shows a typical two component spectra that was quantified by the ratio of the anisotropic and isotropic signals. The isotropic component, associated with a mobile nitroxide free radical, increases during prolonged exposure to all solvents used in the study. The maximum increase (of 43%) was observed in 1,4-dioxane. Based on these and previous studies we suggest that prolonged exposure of the enzyme to these solvents provokes a cascade of events that could induce substrates to adopt different binding conformations. This is the first EPR study of the motion of an active-site spin label during prolonged exposure of an enzyme to organic solvents ever reported.

## 1. Introduction

Enzymes have been successfully employed to catalyze a number of transformations and chiral resolutions of biological and industrial importance in organic solvents [1,2,3,4,5,6,7,8,9,10]. In non-aqueous media autolysis (in the case of proteases) is prevented and the thermostability of the biocatalyst is increased [8,11,12]. However, during prolonged exposure to organic solvents most enzymes loose from 50 to 90% of their enzymatic activity [13]. To shed more light to this problem, we decided to study the active-site pocket environment of the serine protease subtilisin Carlsberg directly, during prolonged exposure to 1,4-dioxane (diox), tetrahydrofuran (THF), acetonitrile (ACN) and hexane (hex). Specifically, the enzyme was spin labeled at its active site with a nitroxide spin label (4-ethoxy-fluorophosphinyloxy-TEMPO)—A technique referred to as “active-site spin labeling” (ASSL), and its movement inside the active site was followed by electron paramagnetic resonance spectroscopy (EPR). ASSL has become a common and powerful technique to extract dynamic and structural data of enzymes and proteins [14,15]. A simple one component EPR spectrum is usually observed when the nitroxide side chain does not interact with the protein side groups. However, a more complex two or more component spectrum is generally observed when the spin label is in the active site, and the nitroxide side chain interacts with the active-site residues side groups. The results here presented show the classical two component spectra previously observed with similar spin labels and enzymes [14,16,17,18,19]: A rigid and a mobile component. The rigid component diminishes considerably while the mobile one increases when the two enzyme preparations here used (the lyophilized powder and the PEG_5000_ chemically modified enzyme) are stored in the solvents for 96 h (except for the PEG-enzyme in ACN were the mobile component predominates during the storage experiments). The two components system is characterized by different spectral features corresponding to isotropic and anisotropic movements of the active site spin label side chain. We used the ratio of these two components to understand what a substrate might actually experience during prolonged exposure to organic solvents, and therefore help us understand the cause for the gradual loss of enzymatic activity observed. 

## 2. Results and Discussion

**Figure 1:**
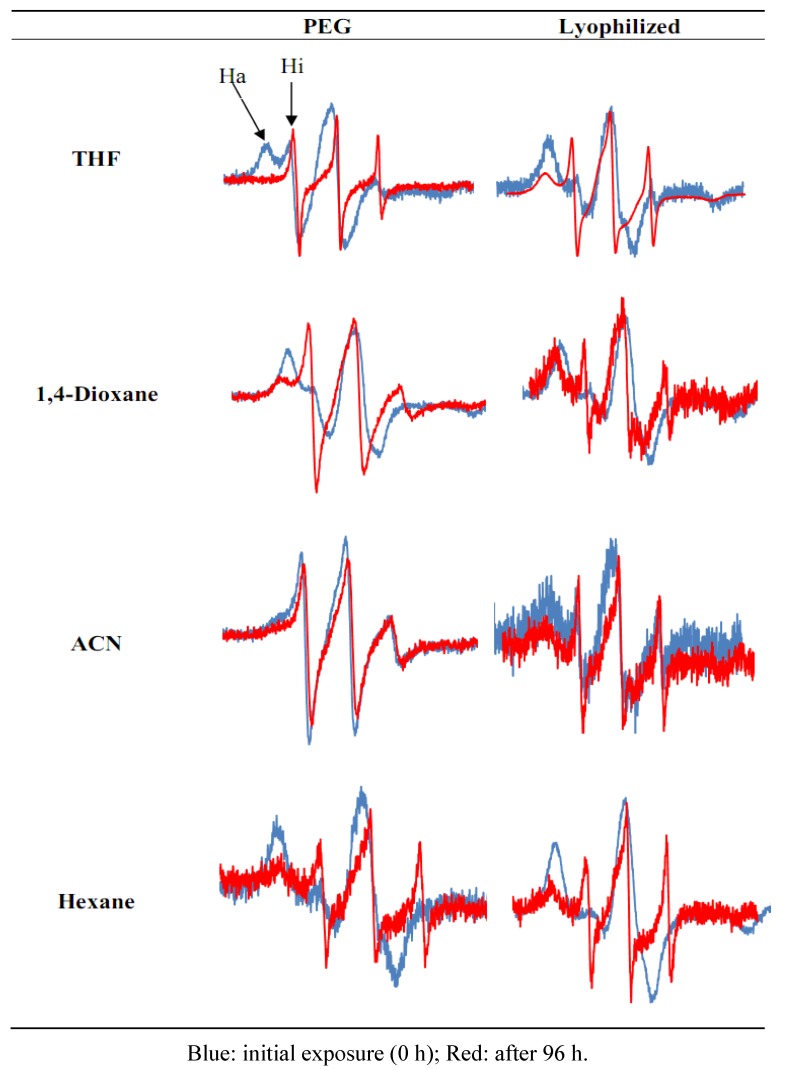
EPR spectra of both enzyme preparations in all solvents.

Loss of initial enzyme activity during prolonged exposure to organic media has been reported by us and others [13,20,21,22,23]. Studies geared to determine the reasons for this phenomenon have generally concluded that this was not the result of partial denaturation, structural changes or aggregation of the enzyme molecules in organic solvents. However, a decrease in the overall enzyme flexibility was observed [24], and a decrease on both K_cat_ and K_M_ suggested subtle changes in the active site pocket [20]. A follow-up fluorescence spectroscopic study recently reported by us suggests that the polarity of the enzyme active-site is affected during prolonged exposure to organic solvents, and points out that this might force substrates to adopt slightly different binding orientations which could result in the observed decreased enzymatic activity [25]. The goal of the present study was to gain a deeper understanding of the effect of prolonged exposure to organic solvents on the active site environment of our model enzyme, subtilisin Carlsberg. To accomplish this, we measured the freedom of rotation of a nitroxide active site spin label by EPR spectroscopy as a function of time of exposure to organic solvents (acetonitrile, hexane, 1,4-dioxane and tetrahydrofuran). The study was completed with the lyophilized powder (for which much kinetic and structural data is available) and with the more active and enantioselective PEG_5000_-chemically modified enzyme. EPR spectroscopy is frequently employed to learn about the dynamics of enzymes suspended in non-aqueous media, and in most cases such information is extracted from a characteristic two component spectra similar to ours [18,19,26]. The components are usually explained as corresponding to spin labels having different freedoms of rotation within the same sample (isotropic and anisotropic motions) [27]. Although information about the flexibility of enzymes and proteins can be extracted [14,15], a more in depth and thorough analysis is required. However, variations in the percent of isotropic and anisotropic components could be due to any of the following: (a) changes in enzyme flexibility, (b) structure, or (c) reorientation of the active site spin label. Our EPR data, presented on Figure 1, shows that in most solvents the isotropic component (mobile one, Hi) increases over time (initial exposure to organic solvents in blue and red after 96 h). This could be interpreted as increased enzyme flexibility (or two enzyme populations were the more flexible one predominates). But as mentioned above, a H/D exchange study reported by us of the same enzyme and solvents systems revealed that actually the enzyme became rigid over time of exposure to organic solvents, and a subsequent study by FTIR and CD spectroscopy demonstrated that the enzyme remains structurally defined in organic media over prolonged periods of exposure. From those results we rule out the possibility that the EPR data reflects mayor global flexibility or structural changes as possible causes for the observed activity loss. Localized dynamic changes of the active site residues could have been undetected by our previous studies, and these could affect the anisotropy of the free radical, however it is unlikely that such an effect would not influence the dynamics of the entire enzyme, and as mentioned above, our H/D dynamic study showed that the enzyme is less dynamic after 96 h of exposure to organic solvents. We therefore propose that the isotropic and anisotropic components come about from different binding orientations of the spin label, which could result from reorientation of the active site residues (due to changes in the polarity of the active site and the enzyme fold–as previously suggested by us) or dilatation of the active site cavity. By simply measuring the high of both isotropic and anisotropic peaks, and using the relationship Hi/(Hi+Ha) (Hi and Ha are the high of the isotropic and anisotropic peaks, respectively), the percentage of both isotropic and anisotropic components was recently determined [18]. Please note that a more comprehensive study would be required to obtain reliable and meaningful dynamic data. We used this simple approach to estimate the fraction of both isotropic and anisotropic components on all spectra, in an effort to understand what an actual substrate might experience during prolonged exposure to organic solvents that results in the observed enzymatic activity loss. 

**Figure 2:**
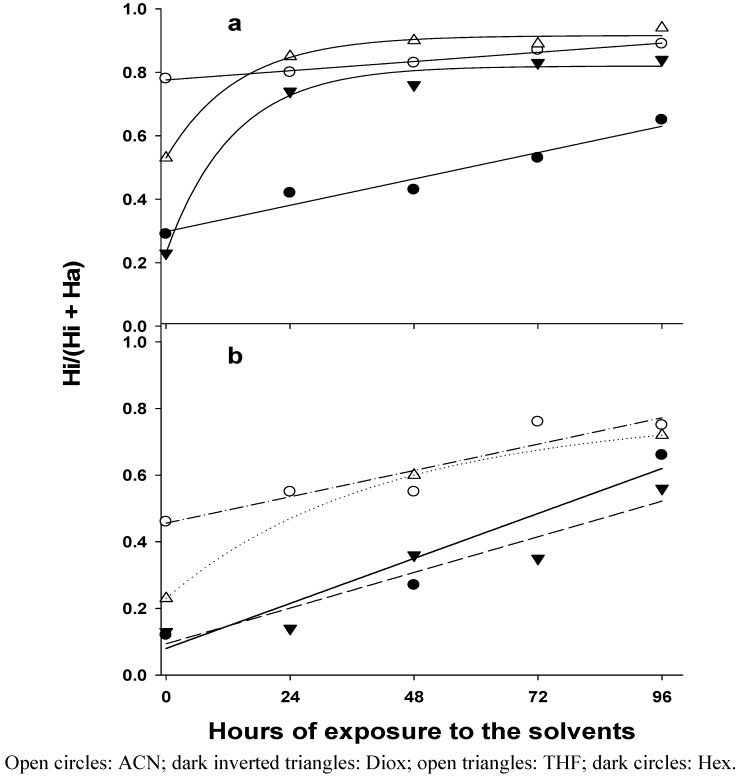
Hi/(Hi+Ha) *vs.* hours of storage of (**a**) PEG preparation; (**b**) lyophilized preparation.

Our data shows some clear and interesting trends. First of all, the ratio Hi/(Hi+Ha) indicates that the rigid component predominates when both enzyme preparations are initially exposed to the solvents (Table 1, and Figure 2 panels A and B). This can also be appreciated visually (Figure 1). In all cases, the mobile component increased during exposure to the solvents, and a larger increase from initial exposure (0 h) is observed in the case of the low dielectric constant solvents.

**Table 1:** EPR data at 0 and 96 hours of incubation in the solvents. Hi and Ha calculated from the high (from baseline) of the isotropic and anisotropic peaks.

	Hi/(Hi/Ha)										
	Hexane			Acetonitrile			1,4-dioxane			THF	
Hours	PEG	Lyo		PEG	Lyo		PEG	Lyo		PEG	Lyo
0	0.29	0.12		0.78	0.46		0.23	0.13		0.53	0.23
24	0.42			0.80	0.55		0.74	0.14		0.85	
48	0.43	0.27		0.83	0.55		0.76	0.36		0.90	0.60
72	0.53			0.87	0.76		0.83	0.35		0.89	
96	0.65	0.66		0.89	0.75		0.84	0.56		0.94	0.72

**Figure 3:**
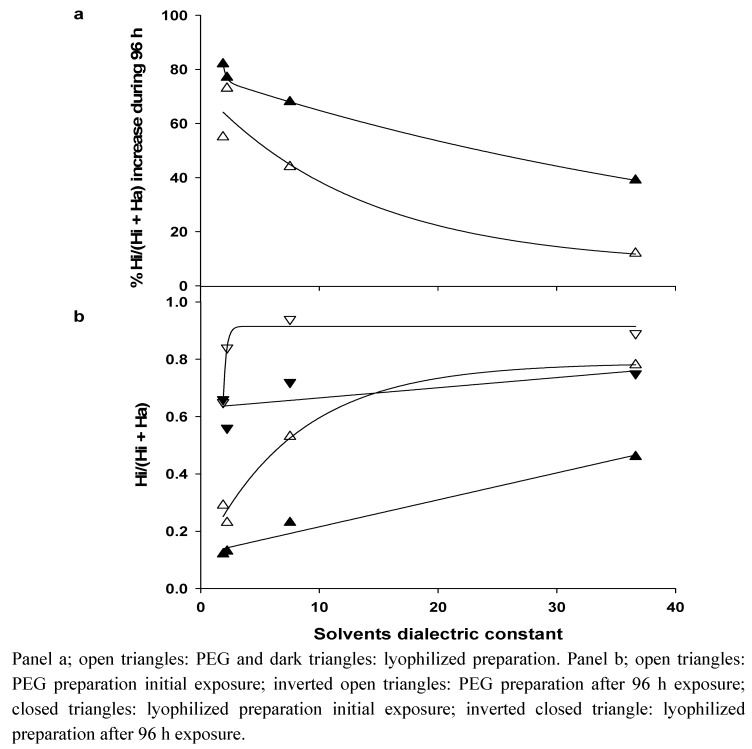
(**a**) % Hi/(Hi+Ha) increase during 96 h *vs.* the solvents dielectric constants; (**b**) Hi/(Hi+Ha) ratio *vs.* the dielectric constants of the solvents.

This becomes more evident in a plot of the solvents dielectric constants *versus* the % increase in Hi/(Hi+Ha) over time (Figure 3a). A trend between Hi/(Hi+Ha) and the solvents dielectric constant is also observed initially (but not after 96 h, Figure 3b). Although more solvents are needed to make an assertion, it seems that in polar aprotic solvents there is already a high % of mobile component before incubation, and prolonged exposure does not increase this ratio as much as in the case of non-polar aprotic solvents. No correlations between the enzyme initial rates (Vs) or the enzyme enantioselectivity and this parameter (Hi/(Hi+Ha)) are observed with either preparation before or after prolonged exposure to the solvents. Table 2 shows the enzyme initial rates (Vs) and enantioselectivity (E) measured initially (0 h) and after prolonged exposure (96 h) to the solvents. It is worthwhile noticing that (a) the mode of enzyme preparation and the solvents have a significant effect on enzyme activity; (b) enantioselectivity is more solvent-dependent than enzyme-preparation-dependent, (c) activity loss during exposure to the solvents for 96 h is observed with all solvents and with both enzyme preparations, and (d) enzyme enantioselectivity seems to be unaffected by the 96 h exposure-period (Table 2). 

**Table 2:** Kinetic data obtained with both preparations in all solvents.

Storage for		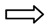	0 h		96 h	
		Vs (mmol/min.mg)	E	Vs (mmol/min.mg)	E
Hex	Lyo	0.016 ± 0.007	12 ± 2	0.0018 ± 0.00006	10 ± 1
	PEG	0.47 ± 0.01	7.4 ± 0.1	0.25 ± 0.01	6.8 ± 0.1
ACN	Lyo	0.0035 ± 0.0009	4.6 ± 0.7	0.00015 ± 0.00007	4.7 ± 0.7
	PEG	0.077 ± 0.002	5.1 ± 0.1	0.011 ± 0.0001	5.2 ± 0.04
THF	Lyo	0.025 ± 0.001	34 ± 5	0.006 ± 0.001	21 ± 3
	PEG	0.62 ± 0.01	36.7 ± 0.4	0.095 ± 0.007	32.1 ± 0.2
Diox	Lyo	0.032 ± 0.002	30 ± 9	0.0027 ± 0.0001	17 ± 1
	PEG	0.6 ± 0.1	21.2 ± 0.3	0.15 ± 0.01	16 ± 1

In a recent theoretical study we demonstrated that during initial exposure of this enzyme to ACN an equilibrium is established between the inter protein water molecules and the bulk of the solvent [28]. On a separate fluorescent spectroscopic study, we also show that prolonged exposure to organic solvents of the same enzyme alters the micro environment of the active site, and shifts the emission maxima of an active site fluorophore inhibitor to lower wavelengths [25]. This suggested to us that prolonged exposure to organic solvents alters the polarity of the active site which could in turn induce the substrate (or active site fluorophore inhibitor) to reorient itself into a new binding conformation. It is believed that water molecules bound deep on the enzyme core and on its surface are essential for (and therefore determine) optimum enzyme activity and enantioselectivity. One can then argue that these observations are entirely related to a hydration effect. A control EPR experiment reported on a different study by us, in which the water activity of the sample was kept constant (by the addition of a hydrated salt mixture to the dioxane + lyophilized enzyme), also revealed a slight increase in the mobile component during incubation [20], however not as much as when the enzyme was incubated without added water [20]. It seems that under this last experimental condition enzyme hydration in organic solvents is quickly pre-equilibrated (during the pre-equilibration step–prior to measure the EPR spectrum), and the spin label acquires its most preferable binding orientation (and mobility). A control experiment in buffer shows primarily the mobile component (95%), which is expected for an enzyme that is in solution (unlike the enzyme in pure organic solvents that is a suspension). EPR studies of proteins and enzymes using similar nitroxide free radicals (TEMPO) as us, generally reported similar data when the free radical is located in (a) highly flexible or (b) unrestricted environment [14,15,16,17]. To rule out that in our case the mobile component was not due to bleaching of the free radical from the enzyme, we washed the lyophilized enzyme preparation after 96 h of exposure to 1,4-dioxane and recorded the EPR of the wash and that of the re-suspended enzyme. No free radical was detected on the wash and the characteristic two component spectrum was observed after re-suspending the enzyme. In short, the results here presented are in agreement with previous studies suggesting that the binding orientations of a substrate before and after incubation are different. The two binding model is also supported by EPR studies using a similar nitroxide free radical bound to different regions of a proteins, which show similar spectra as ours [14,15,16,17]. To the best of our knowledge those spectra have always been interpreted as corresponding to free radical side chains having different degrees of mobility (hence two distinct populations). Perhaps, as the theory on this field matures, these two populations might be found to be related to a gradual transition in the mobility of the free radical side chain, In any case, no direct correlation was observed between the degree of isotropicity of the free radical [determined by the ratio Hi/(Hi + Ha)] to the activity of the enzyme before or after prolonged exposure to organic solvents. Although comparing the results within each preparation and solvent suggest that slow mobility [lower Hi/(Hi + Ha) ratio] is associated to an active site “environment” that promotes high enzymatic activity (Table 1 and Table 2). However, this trend is not observed between preparations or solvents. For example, the most active PEG preparation shows higher % of the mobile component, while the less active lyophilized preparation shows higher % of the rigid component on all solvents (Table 1). This realization highlights that enzyme activity must be a function of several variables, such as the solvents physicochemical properties, the preparation of the enzyme catalysts and its structural integrity and flexibility–some of which can be affected to different degrees upon prolonged exposure to organic media. Nevertheless, our data shows a relationship between the active site environment and the dielectric constants of the solvents, and how this environment is affected from prolonged exposure of the enzyme to the solvents (Figure 3). The mobility of the free radical increases as the dielectric constant of the solvents increases (promoting high % of the most mobile component). On the other hand, during 96 h of exposure to these solvents, the increase in mobility observed is less pronounced in high-dielectric constant solvents (the % diference between the isotropic components is less Figure 3a). One explanation for this observation could be that in high dielectric constant solvents such as acetonitrile, the % of mobile component (isotropic) is initially close to its maximum, and prolonged exposure to the solvent does not increase this % that much. 

## 3. Experimental

### 3.1. Materials

The enzyme, subtilisin Carlsberg (EC 3.4.21.14) was purchased as a lyophilized powder from Sigma-Aldrich (St. Louis, MO, USA). *Sec*-Phenethyl alcohol and the solvents (anhydrous form–water content below 0.005%) were also purchased from Sigma-Aldrich (St. Louis, MO, USA). Polyethylene glycol (5 kDa) was purchased from Nectar Therapeutics Limited (Huntsville, AL, USA). Vinyl butyrate was purchased from TCI America (Portland, OR, USA). The spin-label, 4-ethoxyfluorophosphinyloxy-TEMPO was purchased from Toronto Research Chemicals (North York, ON, Canada). Sephadex PD-10 desalting columns were purchased from Amersham Biosciences (Piscataway, NJ, USA).

### 3.2. Enzyme Preparation (lyophilisation and PEG’s)

Subtilisin Carlsberg was PEGylated with 10 fold molar excess of 5 kDa PEG in sodium borate buffer (pH 9.2). The PEGylation was allowed to proceed with constant stirring for 15 min at 20 °C and then 3 h at 4 °C. After this the pH of the reaction mixture was reduced to 5.5 with dilute HCl to stop the reaction and the solution was dialyzed for 24 h in 25 kDa cutoff dialysis bag. The dialyzed solution was finally lyophilized for 48 h. The degree of PEGylation in terms of number of PEG molecules per molecule of SC was found to be approximately 4, as determined by the method of Habeeb *et al.* [29].

### 3.3. Spin Labeling

Subtilisin Carlsberg was spin labeled at the active site Ser-221 with 4-ethoxyfluorophosphinyloxy-TEMPO. A 10-fold molar excess of free radical (1.0 M stock solution in chloroform) was used for the labeling, which was carried out in sodium acetate buffer (0.1 M) pH 5.5 at room temperature. After the reaction, the remaining free radical was removed using Sephadex G25 desalting columns. The spin labeled enzyme free of any unbound spin label was thereafter lyophilized and used for the EPR analyses. Active site titration using a published protocol [30] revealed a 100% reduction on the number of enzyme-active-sites. This was accompanied also by complete loss of enzyme activity, which demonstrated that indeed, spin labeling took place at the active site. Organic solvents dried overnight over molecular sieves were used for the EPR analysis. The incubation of labeled enzyme in organic solvents was carried out at 25 °C. All EPR spectra were recorded at room temperature on a Bruker EMX EPR spectrometer with a microwave power of 2.0 mW, a microwave frequency of 9.7 GHz and a sweep width of 100 G (resolution 1024 points). Every spectrum was obtained from a minimum of 500 scans which took approximately 1 h. A control experiment was conducted in all solvents here studied to assure that after the 96 h enzyme storage period there was no free spin label in the solution. This was accomplished by washing the suspended enzyme (after 96 h of exposure) with the corresponding solvents, re-suspending it and then the EPR of the enzyme and that of the wash recorded. Neither samples showed free spin label (the EPR spectra of the enzyme suspension showed two components, and that of the wash did not reveal any spin label–data not shown). Additionally, a sample (in THF) was washed with chloroform and re-suspended in the original solvents. Although this procedure is known to cause partial enzyme denaturation the obtained EPR spectra showed a similar two component spectra–data not shown. 

### 3.4. Active Site Spin Labeling (ASSL)

Site directed spin labelling (SDSL) is emerging as a new tool for determination of structure and conformational dynamics of proteins [31]. The basic strategy of SDSL is to introduce a paramagnetic nitroxide side chain at a specific site in a protein sequence and to analyse the EPR spectrum of the spin labeled protein in terms of environmental parameters that characterise the site in protein fold. Changes of macromolecular configuration influencing the surroundings of a covalently attached spin label are reflected in the label’s degree of immobilization, which can be given a quantitative expression in terms of its rotational correlation time (τ) and therefore provide a measure of enzyme flexibility [32]. When the label is freely tumbling in solution, its spectrum consists of three narrow lines of equal intensity. As the rate of tumbling slows the three lines broaden unequally and the spectrum becomes asymmetric, showing peaks corresponding both to isotropic and anisotropic movements of the spin label (Figure 1, PEG-enzyme in THF). A typical EPR of an enzyme spin labeled at the active site (or in a location were the spin label side chain interacts with the enzyme) shows features that correspond to both the isotropic and anisotropic movements of the spin label—This is what we referred to as the “classical two component spectra”. It has been postulated that a slow moving spin label (rigid) generates the anisotropic component, while a freely moving one (or mobile) gives rice to the isotropic part of the spectrum. The ratio of the anisotropic and isotropic pecks (Figure 1, PEG-enzyme in THF) have been used as a measure of the enzyme flexibility by the relationship Hi/(Hi+Ha) [19]. This relationship has been used throughout our studies to determine the freedom of rotation of the spin label bound to subtilisin Carlsberg active site as a function of its exposure to organic solvents.

### 3.5. Enzyme Preparation and Kinetic Measurements

PEGylated/lyophilized Subtilisin Carlsberg powder was activated by lyophilization from a solution of 5 mg/mL in a 20 mM potassium phosphate buffer at pH 7.8, for 24 h. The transesterification reaction between *sec*-phenethyl alcohol and vinyl butyrate was used in all of the kinetic measurements. The product formation was followed by gas chromatography. The GC instruments (HP 6850 and HP 6890, fitted with Chirasil CB columns, FID detectors and He as carrier gas) were calibrated with the chiral ester products of the reactions. The product peak areas and retention times were the same in the presence or absence of the substrates. The substrates (70 mM alcohol and 200 mM vinyl butyrate) and the solvent (1.0 mL) were dried prior to their use. All kinetic experiments were terminated before 10% of the product had been formed. The enzyme enantioselectivity was determined by measuring the initial rates of enzymatic reactions (from plots of product concentration *vs.* time) of both enantiomers [33]. The retention times of the “*R*” and “*S*” products were obtained using samples of the pure enantiomers synthesized from the corresponding alcohol enantiomers. The enzyme enantioselectivity is equal to the ratio: [k_cat_/K_M_]_R_/[k_cat_/K_M_]_S_ = V_R_[S]/V_S_[R] [34].

### 3.6. Enzyme Concentration

Enzyme concentrations were assayed by the BCA method of using bovine serum albumin as standard [35]. 

## 4. Conclusions

Studies have shown that the observed decrease in enzyme activity after prolonged exposure to organic solvents is unrelated to factors such as structural changes or loss of pH memory, and suggest an effect localized in the active site. Recently we proposed a mechanism that involves two possible conformations/orientations of an active site bound fluorophore, in which upon initial exposure to organic solvents, the dominant conformation of the fluorophore is the one that allows for greater interaction with the active site residues [25]. A transition to a more flexible and mobile conformation occurs gradually during storage in organic solvents [25]. This hypothesis is supported further by the results presented here. The observed increase in the freedom of rotation of the active-site bound spin label [increased Hi/(Hi+Ha) ratio] over the 96 h incubation period reflects a change, perhaps in the binding orientation preferred by the active site spin label, in going from a more "rigid" conformation, probably due to stronger interactions with active side residues, to a more "mobile" one, probably due to more interactions with solvent molecules. 

Furthermore, based on these two separate studies we suggest that an equilibrium between inter water molecules and the bulk of the solvent is being established, favoring the more mobile conformation after prolonged exposure to the solvents. It is also clear that there is a trend between the mobility of the active site spin label and the dielectric constant of the solvents—in which high mobility is favored in solvents of high dielectric constants. However, no correlations or trends were observed between the enzyme activity and the parameter here measured. 

## References

[1] Effenberger F., Syed J. (1998). Stereoselective synthesis of biologically active tetronic acids. Tetrahedron.

[2] Jungen M., Gais H. (1999). Application of pig liver esterase catalyzed transesterification in organic media to the kinetic resolution of glycerol derivatives. Tetrahedron-Asymmetry.

[3] Klibanov A.M. (2001). Improving enzymes by using them in organic solvents. Nature.

[4] Lee T., Jones J.B. (1996). Probing the abilities of synthetically useful serine proteases to discriminate between the configurations of remote stereocenters using chiral aldehyde inhibitors. J. Am. Chem. Soc..

[5] Macritchie J.A., Silcock A., Willis C.L. (1997). Enantioselective synthesis of unsaturated α-hydroxy acids. Tetrahedron: Asymmetry.

[6] Roberts S.M., Williamson N.M. (1997). The use of enzymes for the preparation of biologically active natural products and analogues in optically active form. Curr. Org. Chem..

[7] Sanchez V.M., Rebolledo F., Gotor V.  (1999). Candida antarctica lipase-catalyzed doubly enantioselective aminolysis reactions. Chemoenzymatic synthesis of 3-hydroxypyrrolidines and 4-(silyloxy)-2-oxopyrrolidines with two stereogenic centers. J. Org. Chem..

[8] Zaks A., Klibanov A.M. (1988). Enzymatic catalysis in nonaqueous solvents. J. Biol. Chem..

[9] Carrea G., Riva S. (2000). Properties and synthetic applications of enzymes in organic solvents. Angew. Chem. Int. Edit..

[10] DeSantis G., Davis B.G. (2006). The expanding roles of biocatalysis and biotransformation. Curr. Opin. Chem. Biol..

[11] Fitzpatrick P.A., Steinmetz A.C.U., Ringe D., Klibanov A.M. (1993). Enzyme crystal structure in a neat organic solvent. Proc. Natl. Acad. Sci. USA.

[12] Griebenow K., Klibanov A.M. (1995). Lyophilization-induced reversible changes in the secondary structure of proteins. Proc. Natl. Acad. Sci. USA.

[13] Martinez S.G., Alvira E., Cordero L.V., Ferrer A., Montanes-Clemente I., Barletta G. (2002). High initial activity but low storage stability observed for several preparations of subtilisin carslberg suspended in organic solvents. Biotechnol. Progr..

[14] Columbus L., Hubbell W.L. (2004). Mapping backbone dynamics in solution with site-directed spin labeling: Gcn4-58 bzip free and bound to DNA. Biochemistry.

[15] Columbus L., Kálai T., Jekö J., Hideg K., Hubbell W.L. (2001). Molecular motion of spin labeled side chains in α-helices: Analysis by variation of side chain structure. Biochemistry.

[16] Clark D.S. (2004). Characteristics of nearly dry enzymes in organic solvents: Implications for biocatalysis in the absence of water. Phil. Trans. R. Soc. Lond. B.

[17] Lietzow M.A., Hubbell W.L. (2004). Motion of spin label side chains in cellular retinol-binding protein: Correlation with structure and nearest-neighbor interactions in an antiparallel β-sheet. Biochemistry.

[18] Ueji S., Taniguchi T., Okamoto T., Watanabe K., Ebara Y., Ohta H. (2003). Flexibility of lipase brought about by solvents controls its enantioselectivity in organic media. Bull. Chem. Soc. Jpn..

[19] Watanabe K., Yoshida T., Ueji S. (2004). The role of conformation flexibility on enzymes in the diecrimination between amino acid and ester substrates for the subtilisin-catalyzed reaction in organic solvents. Bioorg. Chem..

[20] Castillo B., Bansal V., Ganesan A., Halling P., Secundo F., Ferrer A., Griebenow K., Barletta G. (2006). On the activity loss of hydrolases in organic solvents: II. A mechanistic study of subtilisin carlsberg. BMC Biotechnol..

[21] Castillo B., Pacheco Y., Al-Azzam W., Griebenow K., Devi M., Ferrer A., Barletta G. (2005). On the activity loss of hydrolases in organic solvents: I. Rapid loss of activity of a variety of enzymes and formulations in a range of organic solvents. J. Mol. Catal. B-Enzym..

[22] Castillo B., Sola R.J., Ferrer A., Barletta G., Griebenow K. (2008). Effect of peg modification on subtilisin carlsberg activity, enantioselectivity, and structural dynamics in 1,4-dioxane. Biotechnol. Bioeng..

[23] Fernandez J.F.A., Halling P. (2002). Operational stability of high initial activity protease catalysts in organic solvents. Biotechnol. Progr..

[24] Fasoli E., Ferrer A., Barletta G.L. (2009). Hydrogen/deuterium exchange study of subtilisin carlsberg during prolonged exposure to organic solvents. Biotechnol. Bioeng..

[25] Bansal V., Delgado Y., Fasoli E., Ferrer A., Griebenow K., Secundo F., Barletta G.L. (2010). Effect of prolonged exposure to organic solvents on the active site environment of subtilisin carlsberg. J. Mol. Catal. B-Enzym..

[26] Watanabe K., Yoshida T., Ueji S. (2001). Optimum conformational flexibility of subtilisin to maximixe the enantioselectivity for subtilisin-catalysed transesterification in an organic solvent with an addition of dimethyl sulfoxide. Chem. Commun..

[27] Guo Z., Cascio D., Hideg K., Kálái T., Hubbell W.L. (2007). Structural determinants of nitroxide motion in spin-labeled proteins: Tertiary contact and solvent-inaccessible sites in helix g of t4 lysozyme. Protein Sci..

[28] Cruz A., Ramirez E., Santana A., Barletta G., Lopez G. (2009). Molecular dynamic study of subtilisin carlsberg in aqueous and nonaqueous solvents. Mol. Simulat..

[29] Habeeb A.F.S.A., Cassidy H.G., Singer S.J. (1958). Molecular structural effects produced in proteins by reaction with succinic anhydride. Biochim. Biophys. Acta.

[30] Schonbaum G.R., Zerner B., Bender M.L. (1961). The spectrophotometric determination of the operational normality of an alpha-chymotrypsin solution. J. Biol. Chem..

[31] Hubbell W.L., Cafiso D.S., Altenbach C. (2000). Identifying conformational changes with site-directed spin labeling. Nat. Struct. Biol..

[32] Hamilton C.L., McConney H.M. (1968). Spin Labels. Structural Chemistry and Molecular Biology, Rich, A., Davidson,N.R., Eds..

[33] Griebenow K., Laureano Y., Santos A.M., Montanez Clemente I., Rodriguez L., Vidal M.W., Barletta G. (1999). Improved enzyme activity and enantioselectivity in organic solvents by methyl-β-cyclodextrin. J. Am. Chem. Soc..

[34] Fersht A. (1985). Enzyme Structure and Mechanism.

[35] Smith P.K., Krohn R.I., Hermanson G.T., Mallia A.K., Gartner F.H., Provenzano M.D., Fujimoto E.K., Goeke N.M., Olson B.J., Klenk D.C. (1985). Measurement of protein using bicinchoninic acid. Anal. Biochem..

